# Audit of bacteraemia management in a university hospital ICU

**DOI:** 10.1186/cc13547

**Published:** 2014-03-17

**Authors:** V De Santis, MG Gresoiu, A Peter, R Wilson, M Singer

**Affiliations:** 1 University College London Hospital, London, UK

## Introduction

The optimal duration of antibiotic treatment in critically ill patients remains a subject of debate. In our multidisciplinary ICU, a short course of antibiotic monotherapy (5 to 7 days) is generally used as a strategy to treat bacteraemia, unless specifically indicated otherwise (for example, endocarditis, osteomyelitis). We aimed to determine the impact of this strategy on antibiotic resistance patterns and patient outcomes compared with a similar exercise we conducted in 2000 [1].

## Methods

We conducted a retrospective study of all patients with bacteraemia or fungaemia (community-acquired, hospital-acquired, and ICU-acquired) treated in our university hospital ICU over a 6-month period (December 2012 to May 2013). We compared this against data from blood culture-positive patients admitted between February and July 2000. Information was collected on bacteraemia episodes, causative pathogens, antimicrobial resistance patterns, antibiotic use and duration, and patient outcomes. Notably, our ICU admits many immunosuppressed patients (for example, haemoncology).

## Results

**Figure 1:**
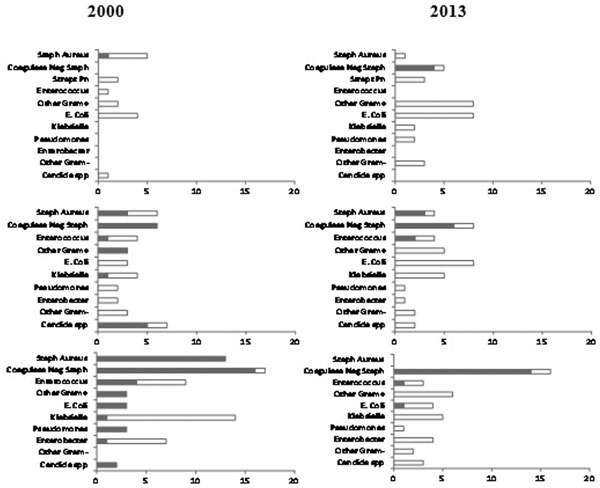
**Microorganisms isolated in each bacteraemia group**. Left panel, 2000; right panel, 2013. Top panel, community-acquired bacteraemia; Middle panel, hospital-acquired bacteraemia; lower panel, ICU-acquired bacteraemia. Shaded areas represent numbers of methicillin-resistant strains for S. aureus and coagulase-negative staphylococci; vancomycin-resistant strains for Enterococcus spp.; multidrug-resistant strains for Gram-negative pathogens; and fluconazole-resistant strains for Candida spp.

Table 1 presents demographics and incidence of bacteraemia. Antimicrobial resistance remained low in the 2013 cohort with few multi-resistant Gram-negative organisms, few fungaemia episodes and a marked decrease in methicillin-resistant *Staphylococcus aureus *(MRSA) (Figure 1). The number of relapses and breakthrough bacteraemias remained low.

**Table 1:** Demographics and incidence of bacteraemia

Variable	2000 cohort	2013 cohort
Total ICU population	713	1,318
Patients with bacteraemia	91	87
Episodes of bacteraemia	102	113
Community-acquired bacteraemia	13	37
Hospital-acquired bacteraemia	28	39
ICU-acquired bacteraemia	60	37
Hospital mortality	45%	32%
Monotherapy treatment (%); duration median (IQR)		
Community-acquired bacteraemia	57%; 6 (5 to 6)	65% 5 (3 to 5;
Hospital-acquired bacteraemia	78%; 6 (5 to 8)	63%; 5 (4 to 7)
ICU-acquired bacteraemia	80%; 5 (5 to 7)	62%; 4 (3 to 6)

## Conclusion

A strategy of short-course antibiotic monotherapy is associated with low breakthrough and relapse rates and a low rate of antibiotic resistance.
